# Can optimal caching models predict changes in diurnal feeder use patterns in black-capped chickadees (*Poecile atricapillus*)?

**DOI:** 10.1098/rsos.261406

**Published:** 2026-10-28

**Authors:** Mechelle Goulart, Ipshita Gayen, Nathan Hobbs, Jan J. Wijmenga, Kimberley J. Mathot

**Affiliations:** Department of Biological Sciences, University of Alberta, Edmonton, Alberta, Canada

**Keywords:** diel activity, food storage, optimal daily routines, passerine, scatter hoarding, small bird in winter

## Abstract

Many birds collect and store food for later use, a behaviour known as caching. Theoretical models predict that caching effort is concentrated in a single peak during the day, though the precise timing of that peak depends on multiple factors. We studied diurnal patterns of feeder used in a population of 50 individually marked black-capped chickadees (*Poecile atricapillus*) in late summer. We used total daily feeder visits (1 visit ≈ 1 sunflower seed) as a proxy for caching intensity because daily feeder visits (range 11–339 feeder visits per bird per day) often exceeded the required intake of about 45 sunflower seeds for chickadees at this time of year, consistent with a wide range of caching effort. Patterns of feeder use were always bimodal, and increases in hourly feeder visits occurred throughout the day with increased total daily feeder visits, however, the greatest relative increase in feeder visits was during midday. Post hoc analyses revealed that this population-level effect was driven by individual differences; some chickadees increased morning feeder visits with increasing total daily feeder visits, while others increased midday or end-of-day feeder visits. Future work should investigate drivers of these individual differences, including the role of interference competition.

## 1. Introduction

Each day, diurnal birds must build up enough fat stores during the daytime foraging period to meet their metabolic demands to survive the overnight, non-foraging period. For small diurnal birds, including black-capped chickadees (*Poecile atricapillus*), the daily cycle of mass gain during foraging and overnight fasting can exceed 10% of the total body mass [1–4]. Understanding how small birds allocate foraging effort during the day to meet their energetic demands under changing environmental constraints has been a major area of interest in the field of behavioural ecology [5–8] and the focus of several formal mathematical models [9–15].

Models of optimal daily foraging generally predict bimodal foraging patterns, with a morning peak in foraging to mitigate increased starvation risk following the overnight fast and an end-of-day peak predicted as birds build up fat stores for the coming overnight, non-foraging period [10,13–15]. Delaying mass gain for the upcoming overnight fast is thought to minimize the time period over which birds experience high mass-dependent predation risk [16]. However, the challenges of acquiring enough energy during the foraging period to survive both the day and night change seasonally. During winter, energetic demands are highest, owing to the higher costs of thermoregulation, which also coincides with the lowest natural food availability and shortest daylength available to acquire food, at least for diurnal species [5,14,15]. To cope with these challenges, some birds build up food stores in late summer and early autumn, called caches, when food is still abundant, energetic costs are comparatively low, owing to milder temperatures, and longer daylengths provide a relatively longer foraging window [17]. These long-term caches provide a predictable food source that can be used throughout the winter, and because they are held externally, they do not impose mass-dependent costs on birds, as is the case with body fat stores [16,18]. A number of models have been developed to investigate diurnal investment in caching, though these models are focused on either short-term caching (i.e. caches retrieved within a day) [18,19] or both short- and intermediate-term caching (i.e. caches retrieved up to several weeks after storage) decisions [15,20,21], rather than the long-term caching that occurs during the late summer/early autumn peak in caching effort. These models of optimal caching predict that caching effort should be concentrated at specific times of the day (see figure 1). Caching is predicted to be concentrated in the morning when there are mass-dependent metabolic costs for activity [15,18,21] and/or there is mass-dependent predation risk [15,21], which makes investment in caching least costly early in the day, before birds have increased fat reserves. Midday peaks in caching are predicted when starvation risk is high at dawn (necessitating building up fat reserves) and predation risk is high at dusk owing to the mass-dependent predation costs [19,20]. Finally, end-of-day peaks in caching are predicted when starvation risk is very high [15] because in this case, caching is too costly until late in the day when sufficient reserves have been built up to reduce starvation risk and/or predation risk declines throughout the day [21], making caching less costly later in the day owing to the decreased predation risk.

**Figure 1.**
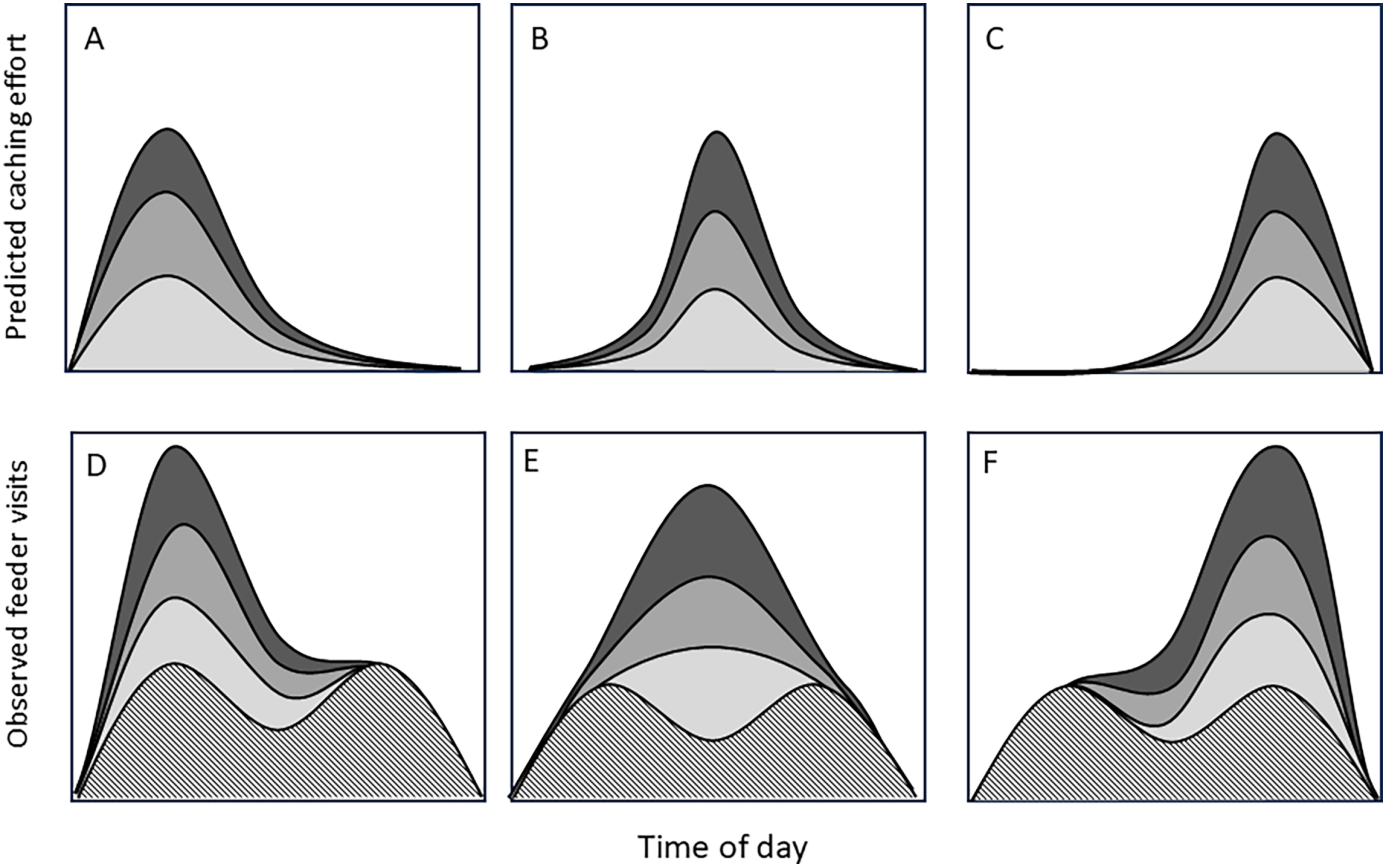
Predictions from models of optimal caching (A–C) and the combined effects of caching (grey-toned areas illustrate three intensities of caching: light grey = low intensity, medium grey = intermediate intensity, and dark grey = high intensity) and eating (hatched areas) on total feeder visits throughout the day assuming that caching does not shift underlying feeder visits for intake (D–F). Caching is generally predicted to be concentrated at specific times of the day. (A) Morning peaks in caching are predicted under conditions where starvation risk is low and/or predation risk is either uniform through the day or decreases through the day [15,18,21], (B) midday peaks are predicted when there are mass-dependent trade-offs between starvation and predation risk [19,20], and (C) end-of-day peaks are predicted when predation risk increases throughout the day [21] and/or starvation risk is high [15]. If we assume that feeder visits for immediate food consumption are bimodal [16] (hatched curve in panels D–F), then the resultant feeder visits from a morning peak in diurnal caching (A) are shown in (D), the resultant feeder visits from a midday peak in diurnal caching (B) are shown in (E), and the resultant feeder visits from an end-of-day peak in diurnal caching (C) are shown in (F).

Black-capped chickadees, hereafter chickadees, are small, non-migratory songbirds that cache extensively [22]. Here, we use data from a marked population of chickadees to evaluate how diurnal patterns of feeder use change with increasing caching intensity, as inferred from total daily feeder visits. The data were collected over a six-week period in late summer, during the time of year when caching is expected to peak [23,24]. At this time of year, chickadees are building up long-term rather than short- or intermediate-term caches, and thus, chickadees are expected to invest heavily in caching despite food being provided ad libitum. Indeed, long-term caching is thought to have evolved specifically to occur at times of the year when food abundance is very high, before seasonal declines in food availability [25,26]. However, we assume that trade-offs between mass-dependent starvation and predation risk that have been identified as shaping diurnal patterns of investment in foraging versus short- or intermediate-term caching can similarly be expected to shape diurnal investment in long-term caching. We found that feeder visitation patterns were always bimodal. Further, chickadees increased hourly feeder visits throughout the day as daily feeder visits increased, with the greatest relative increase in hourly feeder visits occurring midday. We discuss our results in light of existing models of optimal caching as well as previous empirical work and suggest avenues for future research.

## 2. Methods

### 2.1. Field site and study species

This study was conducted at the University of Alberta Botanic Garden (UABG) in Devon, Alberta (53°24′29″N 113°45′24″W), approximately 20 km southwest of the city of Edmonton. The UABG is a 0.97 km^2^ property comprised of manicured gardens (0.32 km^2^) and mixed woodlands (0.65 km^2^). A marked population of chickadees has been maintained at the UABG since Fall 2017 through annual catching and banding efforts. Banding typically occurs between October and December each year, with 100–150 birds caught annually. Annual survival in this population is approximately 60% [27,28].

Upon capture, chickadees are banded with a Canadian Wildlife Services metal band (size 0, 0.045 g) and a unique combination of one standard colour leg band (Avinet Research Supplies, Vynalast XF 2.3 - 2.5 mm bands, 0.02 g) and one colour leg band with an embedded passive integrated transponder (PIT) tag (2.6 mm EM4102 PIT tags; Eccel Technology Ltd, UK, < 0.1 g) for individual identification. The combined mass of these auxiliary markers is ^~^1.5% of the body mass of a chickadee (range approx. 10–12 g). When a PIT-tagged chickadee visits a feeder with a radio-frequency identification (RFID) antenna, the identity of the individual is recorded, along with the date and time of the visit (see below). As this study was conducted between August and September 2025, all of the marked birds included in the study were caught and banded in Fall 2024 or earlier, and were therefore 2nd calendar year birds and older.

Feeders were installed on 31 July 2025 (midday) and baited continuously with black-oil sunflower seeds through to 11 September 2025, a six-week period that was expected to coincide with a seasonal peak in caching based on earlier work in chickadees [23,24]. As our study period coincided with the seasonal peak in long-term caching investment in black-capped chickadees [24], providing ad libitum food was not expected to reduce caching investment as long-term caching has evolved to occur at times of the year when food abundance is very high [25,26]. Conditions during the study period were relatively mild, with average daytime temperatures of 19.27°C (range: 11.77°C–25.17°C) and average overnight temperatures of 11.69°C (range: 2.45°C–16.27°C) [29]. PIT-tagged chickadees were monitored continuously at all eight feeders located in the mixed woodland areas of the UABG. Feeders were separated by at least 270 m, and this spacing was chosen based on previously published winter flock territory sizes [22]. Indeed, in this population, over 50% of birds use a single feeder over the entire season, and those that do use more than one feeder exhibit a strong preference for a single feeder [30]. Each feeder was equipped with a 125 kHz RFID antenna connected to a circuit board (Priority 1 Design, Australia) and powered with a 12 V lead sealed acid battery. The data logging parameters were set to register individual arrival to a feeder with a resolution of 1 second. Data were stored on a standard SD card. Comparisons of observations of leg-banded chickadees against RFID detections confirmed that this system has a 100% detection accuracy; out of 353 video observations of chickadees with leg band PIT tags, 353 visits were registered by the RFID system [31]. Feeders were visited every 7 days to refill sunflower seeds, download the data, and replace the batteries.

### 2.2. Data filtering and processing

Prior to the analyses, we processed the raw RFID data to remove erroneous reads and to collapse repeated lines of data into unique feeder visits (see supplementary material, text S1, for detailed filtering procedures). Following data filtering and processing, our dataset included *n* = 124507 feeder visits by *n* = 50 birds, over *n* = 42 days from 1 August 2025 to 11 September 2025. We used average anchoring of time, which fixes anchor points at the mean Zeitgeber times across the study period [32], to account for variation in daylength over the course of the 42-day study (sunrise range: 05.49–07.00; sunset range: 19.58–21.29) [33]. This method converts observed timestamps to solar radians so that times are standardized relative to sunrise and sunset over the 42-day study period despite the seasonal changes in daylength. In other words, following standardization, sunrise and sunset are expressed as the same times on all days (i.e., the mean sunrise and sunset times over the course of the study), and the intervening hours are scaled as appropriate [see 32 for more details]. As chickadees are diurnal and constrained to foraging approximately between sunrise and sunset (e.g. [6]), this conversion provides unbiased estimates of activity patterns within the daytime foraging window [32]. To do this, we applied the function ‘solartime’ from the package ‘Activity’ [34] to a column listing the timing of each feeder visit with the format ‘year:month:day hour:minute:second’ which converted each visit time to solar time, thus accounting for the changes in daylength within study period.

To model diurnal foraging patterns, we first divided each day into 24 one-hour intervals using the solar converted times (see above); intervals within each day ranged from 0 to 23, where 0 = 00.00–0.59, 1 = 01.00–01.59, and so on. We then calculated the total number of foraging visits per bird per day across all feeders during each 1-hour interval using the average anchored time column. If a bird was present during any hour interval of a given day, the hours where it was not present on the same day were assigned zero visits. Next, we excluded bird days (i.e. unique combination of bird ID and date) where the total number of visits was less than or equal to 10, as that was an insufficient number of visits from which to model diurnal foraging patterns [6,35]. This resulted in the exclusion of 146 out of 1530 bird days or 9.5% of the total datapoints. Thus, our final sample size for analysis was *n* = 33144 hourly feeder visit observations of *n* = 50 birds.

### 2.3. Statistical analyses

All statistical analyses and RFID data processing were executed using the R Statistical Environment [36] using the RStudio interface [37]. We used hierarchical trigonometric models [38] to quantify diurnal foraging patterns and test whether these patterns changed as the intensity of caching changed. These models are well suited for analyses of diel activity patterns when observations have a hierarchical structure [38], such as repeated observations of the same individuals, or across individuals on the same day, as was the case with our data. We constructed our hierarchical trigonometric models [38] with Tweedie error distributions using the glmmTMB function from the *glmmTMB* package [39,40]. Tweedie error distributions are appropriate for continuous data that are constrained to be positive, but with substantial zero inflation [41]. Data on hourly feeder visits were zero inflated because chickadees are diurnal, resulting in several hours with zero feeder visits each day before dawn and after dusk.

We created separate models for two response variables: (i) total hourly feeder visits and (ii) proportion of total daily visits occurring within each hour (i.e. visits per hour divided by visits per day). The latter response variable was included because higher values of total daily feeder visits would necessarily be associated with greater amplitudes of feeder visits. Modelling the proportion of feeder visits per hour allowed us to compare the daily distribution of feeder visits independent of changes in total feeder visits. For all models, individual ID and day were included as random effects to account for non-independence from repeated measures within individuals and within days.

For both response variables (visits per hour and proportion of total visits per hour), we first evaluated whether diurnal foraging was best explained by a flat, unimodal, or bimodal distribution. The bimodal distribution was best supported for both response variables (see supplementary material, text S2 and table S1). Therefore, we subsequently modelled bimodal diurnal foraging patterns for both response variables and included total daily feeder visits (i.e. the count of feeder visits per bird per day) as a fixed effect in interaction with each of the trigonometric (sine and cosine) terms describing the bimodal foraging pattern [38]. This allowed us to evaluate whether there was support that the diurnal foraging pattern varied as a function of the intensity of feeder use (range 11–339 daily feeder visits), which was assumed to be a useful proxy for caching effort as more than 65% of observations of total daily feeder visits (i.e. 1019 out of 1502 observations of total daily feeder visits) exceeded daily energy requirements (approx. 45 visits per day ≈ 45 sunflower seeds per day; see supplementary material, text S3, for calculation). Total daily feeder visits were centred and standardized prior to the analyses using the ‘scale’ function from the base R package [36]. Differences in diurnal foraging patterns were assessed through visual inspection of plots following recommendations by Iannarilli *et al.* [38].

## 3. Results

We analysed diurnal patterns of feeder visitation for *n* = 50 chickadees across 42 days between 1 August 2025 and 11 September 2025. The average number of daily feeder visits per individual was 82.89 (range 11–339). Individual chickadees were detected at our feeders for a mean of 30 days each (range 4–42 days).

There was strong support that the overall pattern of diurnal foraging was bimodal (supplementary material, table S1). Further, diurnal patterns of feeder use varied as a function of total daily feeding visits for both hourly feeder visits (supplementary material, table S2; figure 2A) and the proportion of feeder visits per hour (supplementary material, table S3; figure 2B). Visual inspection of model predictions revealed that the timing of the morning and end-of-day peaks of hourly feeder visits were broadly similar for all levels of total feeder use, occurring at approximately 08.30 and 15.00, respectively (figure 2A). The amplitude and breadth of both the morning peak and end-of-day peaks increased with increasing total daily feeder visits, with the midday lull becoming less pronounced. The distribution of the proportion of total feeder visits indicated that as the intensity of feeder use increased, feeder visits increased relatively more during midday, with a lower proportion of total daily feeder visits occurring in the morning and end-of-day (figure 2B). The timing of feeder visits also shifted as total daily feeder visits increased; morning foraging commenced sooner, though end-of-day foraging ended at a similar time regardless of total feeder visits (figure 2).

**Figure 2.**
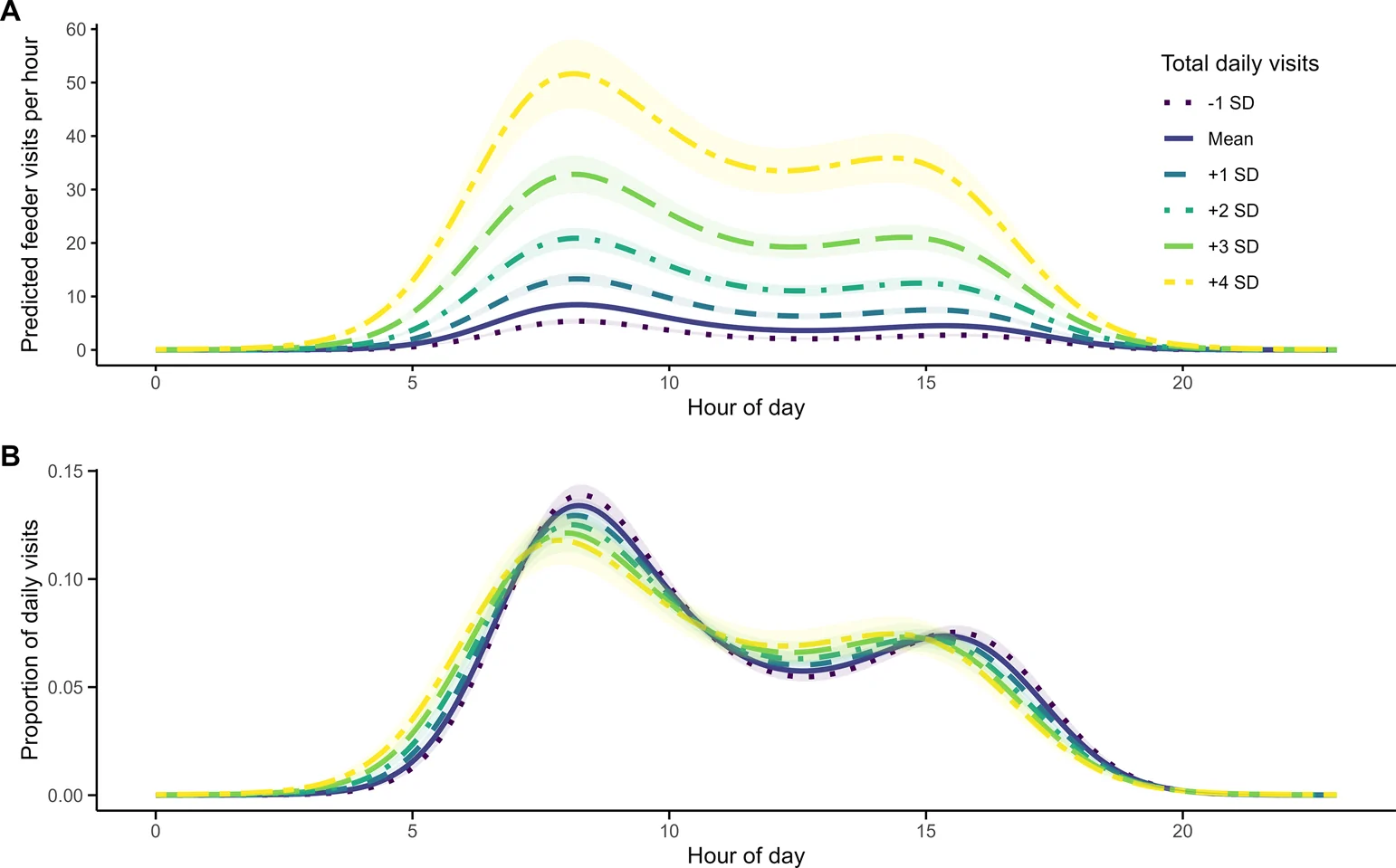
Illustration of the effect of increasing total daily feeder visits on diurnal patterns of (A) hourly feeder visits and (B) the proportion of daily feeder visits occurring within each hour. Note that analyses were carried out using continuous variation in total daily feeder visits per bird; however, for illustrative purposes, model predictions for six levels of daily feeder use are plotted that capture the full range of daily feeder visits observed in our study. For reference, 1 s.d. below the mean corresponds to 29 feeder visits per day, while 4 s.d. above the mean corresponds to 331 feeder visits per day. Lines reflect predictions derived from the trigonometric models, and the shaded areas around each line represents the 95% CI. Predictions and 95% CI were calculated using the ‘emmeans’ function from the ‘emmeans’ package in R [42].

## 4. Discussion

Models of optimal caching predict that caching effort will primarily occur in the morning [15,18,21] or midday [19,20], though end-of-day peaks are predicted when expected intake is below requirements [15] and/or predation risk decreases from morning to end-of-day [21]. We evaluated support for shifts in diurnal patterns of feeder use with changing caching intensity in 50 individually marked black-capped chickadees over a six-week period between August and September 2025. Patterns of feeder use were always bimodal. We also found that when the total number of daily feeder visits increased, increases in hourly feeder visits occurred throughout the day, though the greatest relative increase in feeder visits was midday. Below, we discuss these results in light of existing models of optimal caching and propose directions for future research.

We were interested in evaluating how chickadees alter diurnal patterns in feeder use as caching intensity increases. An important limitation of this work is that we did not track birds after feeder visits, and therefore, cannot determine whether any given feeder visit was for immediate food consumption or caching. However, we interpret increased total daily feeder visits as indicative of increased caching for several reasons. First, chickadees without access to supplemental food exhibit a strong preference for caching seeds over insects, larvae and berries [24]. By providing predictable, ad libitum access to seeds at feeders, we expect that chickadees would preferentially obtain seeds for caching from feeders because this would reduce search time relative to naturally occurring seeds. Further, chickadees retrieve a single sunflower seed per visit (I.G. 2025, unpublished data). Although we do not know to what extent chickadees relied on natural food versus sunflower seeds for consumed food during our study, the average expected energy requirements for chickadees at this time of year is approximately 45 seeds per day (estimated range: 33–61 seeds per day across the range of temperatures observed in this study; see supplementary material, text S3, for calculations). Thus, the range of daily feeder visits in the present study (11–339 visits per bird per day) includes values that fall below daily energy requirements and presumably allow for little or no caching if chickadees use feeders for both caching and eating (e.g. purple dotted lines in figure 2 represents 24 feeder visits per day), up to total daily feeder visits that would allow for high levels of caching, even if chickadees use feeders for both caching and eating (e.g. yellow dot-dashed lines in figure 2 represent 331 feeder visits per day). Thus, we assume that higher total collection of sunflower seeds on the scale observed in the present study correlates with higher total caching, though we recognize that the correlation may not be 1 : 1 depending on whether or how chickadees vary in their use of feeders for direct intake.

We observed bimodal foraging patterns across all levels of total daily feeder use, with a morning peak in feeder use around 08.30 and end-of-day peak in feeder use around 15.00 (figure 2). This pattern is broadly consistent with theoretical predictions based on the assumption that small birds must simultaneously balance predation and starvation risk [10,12,43]. Although chickadees had ad libitum access to black-oil sunflower seeds during our study, this probably does not remove starvation risk as chickadees must still endure overnight fasts [22], and in late summer/early autumn, birds put on less mass prior to the overnight fast compared with winter, when the fast is longer [44,45]. Thus, despite the shorter fast duration at this time of year, birds start the day with depleted reserves which must quickly be replenished. Indeed, other studies providing ad libitum access to food have similarly reported bimodal foraging patterns [46–48].

By contrast, at least two earlier studies in chickadees found that mean feeder visits per hour tended to increase linearly throughout the day, with a marked peak in feeder visits approximately 2 hours before sunset [7,49]. Both of these studies were conducted in winter under conditions of short daylengths and low ambient temperatures between approximately −15°C and 10°C [7,49], and the authors suggested that high energetic demands in winter might require constant feeding to mitigate starvation risk, with no opportunity for a midday lull in foraging [7]. Indeed, our study, conducted in late summer, had longer daylengths and higher daytime temperatures (range: 12°C to 25°C). However, analyses of diurnal foraging patterns in the same population as the present study conducted in winter, under short daylengths and low ambient temperatures (−35°C to 10°C), also observed bimodal foraging patterns in chickadees [50]. Thus, neither longer photoperiods nor milder temperatures are sufficient to explain why we observed bimodality in foraging activity of chickadees in the present study, while at least two earlier works did not [7,49]. Nonetheless, future work explicitly testing the roles of daylength and ambient temperature in shaping diurnal foraging patterns is warranted.

We also found strong support that the distribution of feeder visits throughout the day varied as a function of total daily feeder use, our proxy for caching intensity. Specifically, as total daily feeder use increased, chickadees exhibited increased hourly visits throughout the foraging window (figure 2A), inconsistent with predictions based on a single peak in caching (see figure 1). Analysis of the hourly proportion of total feeder visits revealed that as total daily feeder visits increased, there was a disproportionate increase in feeder visits during the midday lull (figure 2B). This probably occurred because the lower absolute level of feeder visits at this time (see figure 2A) meant that chickadees had greater scope to increase feeder visits midday. We also observed that morning feeder use commenced earlier with increasing intensity of feeder use, though ended at similar times regardless of intensity of feeder use (figure 2A). This is partially consistent with work conducted during winter which similarly found that increased total daily feeder visits by chickadees was achieved by expanding the total foraging window, though this was achieved via both an earlier start and later end to foraging [6].

Our findings that chickadees increased intensity of feeder use throughout the day are not consistent with theoretical models of optimal diurnal caching, which predict that caching intensity should have a single peak either early in the day [15,18,21] (figure 1A), midday [19,20] (figure 1B), or end-of-day [15,21] (figure 1C), depending on a range of factors including starvation risk, variance in foraging success, and temporal patterns in predation risk. Although we are aware of at least one model that predicts constant caching effort throughout the day, the authors state that the model lacks biological realism as it assumes caching decisions will be indifferent to current state, including immediate starvation risk [19]. Indeed, the majority of earlier empirical studies have reported clear peaks in caching effort in the morning [5,51,52], at midday [19] or at end of the day [53], though two studies found no overall time-of-day effect [54,55]. These include studies conducted in both the laboratory [19,52,53,55] and the field [5,51,54], as well as studies where birds had access only to naturally available food [51,54], ad libitum food [52,55], or restricted access to human provided food [5,19,53]. Interestingly, in the study by Boisvert [55], high caching throughout the day arose in a group of *n* = 3 chickadees because one bird had a morning peak, one a midday peak, and one an end-of-day peak, resulting in high caching at the flock-level throughout the day. Individual differences in the timing of caching may explain the patterns observed in the present study as well. Post hoc tests including random slopes in the trigonometric models revealed that individuals exhibited significant variation in the effect of increased feeder visit intensity on diurnal foraging patterns (see supplementary material, text S4, tables S4–S6, and figures S2 and S3); within our population, individual chickadees varied in how diurnal patterns of feeder visits changed under increased total daily feeder use. This may suggest that the relative importance of starvation risk, variance in foraging success, and temporal patterns of predation risk differ for different individuals, leading to different optimal timing of peak caching intensity. Alternatively, individual differences in the timing of peak caching could arise via interference competition [56]. For example, when competing for access to feeders, subordinate birds may shift the timing of peak caching intensity away from the preferred timing of dominant birds as a means of mitigating interference competition. Interference competition is not considered in existing models of optimal caching, which are all optimization models based on single decision makers. However, our work suggests that clumped high value resources, such as feeders, may generate distinct temporal patterns in caching via interference competition. Such resource clumping could also occur naturally, such as if seed bearing trees are rare within a mixed-forest landscape, suggesting that explicit consideration of the role of interference competition in shaping caching decisions is warranted. While the level of competition can easily be controlled in captive studies by varying flock size, field-based studies using feeders are also possible. For example, a study in the same population carried out in the winter found that decreasing the number of feeders per site with ad libitum sunflower seeds from two to one resulted in a reduction in per capita daily feeder visits in chickadees of 50% [57]. This suggests that manipulation of the number of feeders per flock provides a tractable means of manipulating competition levels in free-living populations.

In this study, chickadees consistently exhibited bimodal patterns of feeder visitation. At the population level, increased investment in caching was achieved by increased feeder visitation throughout the daily foraging window (figure 2). Post hoc analyses show that the population-level pattern of high caching throughout the day was driven by different patterns at the level of individuals, with some individuals concentrating feeder visits earlier in the day as total feeder visits increased, and others concentrating effort later in the day. We suggest that interference competition over feeder access might drive among-individual differences in diurnal caching decisions. Future work should explore whether individual differences in temporal patterns of caching are associated with factors expected to influence susceptibility to interference competition, such as dominance, or whether diurnal patterns of caching change when competition is relaxed by providing more feeders per site.

## Supplementary Material



## Data Availability

All the data and R code required to reproduce are available via the Open Science Framework [58]. Supplementary material is available online [59].
