# Supplementary material for: Can optimal caching models predict changes in diurnal feeder use patterns in black-capped chickadees (Poecile atricapillus)?
Source: R Soc Open Sci. 2026 Oct 28;13(10):261406. doi: 10.1098/rsos.261406 (PMC13714374; doi:10.1098/rsos.261406)
Supplement: Supplementary file 1 [file rsos.261406.zip › rsos.261406/rsos261406_si_001.docx]

**Electronic Supplementary Materials (ESM) for:**

**Can optimal caching models predict changes in diurnal feeder use patterns in black-capped chickadees (*Poecile atricapillus*)?**

**DOI: 10.1098/rsos.261406**

Mechelle Goulart^1*^, Ipshita Gayen^1*^, Nathan L. Hobbs^1^, Jan J. Wijmenga^1^, Kimberley J. Mathot^1,†^

^1^Department of Biological Sciences, University of Alberta, Edmonton, Alberta, T6G 2E9

^*^Indicates joint-first authors

^†^Author for correspondence: mathot@ualberta.ca

Contents

[Text S1: Data filtering and processing	3](#_Toc238649680)

[Text S2: Testing for bimodality	5](#_Toc238649681)

[Text S3: Calculation of daily energy requirements	5](#_Toc238649682)

[Text S4: Post hoc tests for random slopes	6](#_Toc238649683)

[Table S1: Model comparisons for hourly feeder detections and proportion of total feeding. For each response variable, we evaluated whether the diurnal pattern of foraging was best modelled using a flat (cathemeral), unimodal, or bimodal distribution. For both response variables, the diurnal patters were best explained by the bimodal model (lowest AIC value).	8](#_Toc238649684)

[Table S2: Full model output for binomial trigonometric model for diurnal pattern in total hourly feeder visits in interaction with total daily feeder visits. The model was fitted with a Tweedie error distribution, and the model dispersion parameter (Φ) was 3.2.	9](#_Toc238649685)

[Table S3: Full model output for binomial trigonometric model for diurnal pattern in the proportion of total daily visits within each hour in interaction with total daily feeder visits. The model was fitted with a Tweedie error distribution, and the model dispersion parameter (Φ) was 0.115.	10](#_Toc238649686)

[Table S4: Full model output for post-hoc test for random slopes in the binomial trigonometric model for diurnal pattern in total hourly feeder visits in interaction with total daily feeder visits. The model was fitted with a Tweedie error distribution, and the model dispersion parameter (Φ) was 2.96.	11](#_Toc238649687)

[Table S5: Full model output for post-hoc test for random slopes in the binomial trigonometric model for diurnal pattern in the proportion of total daily visits within each hour in interaction with total daily feeder visits. The model was fitted with a Tweedie error distribution, and the model dispersion parameter (Φ) was 0.102.	12](#_Toc238649688)

[Table S6: Model comparison between models without vs. models with random slopes for changes in diurnal foraging patterns as a function of total daily feeder visits. Chi^2^ and p-values provide strength of support for the more complex model structure (i.e., random slopes)	13](#_Toc238649689)

[Figure S1. Map of the field site at the University of Alberta Botanic Garden, Alberta, Canada. (53° 24' N, 113° 45' W, 0.97 km^2^). The black dot in the inset map represents the location of the field site within Canada. Garden limits are shown by solid grey lines; dotted grey lines represent walking pathways within the garden. Feeder locations are represented by black circles. West of the garden, the black lines represent Alberta Highway 60. The grey shaded area represents the public visitors’ area, which includes the managed horticulturist gardens but also mixed forest areas. Map created by Josué Arteaga-Torres, and is used with permission.	14](#_Toc238649690)

[Figure S2 : Illustration of the among-individual differences of the effect of increasing total daily feeder visits on diurnal patterns hourly feeder visits. Note that analyses were carried out using continuous variation in total daily feeder visits per bird, however, for illustrative purposes, model predictions for 4 levels of daily feeder use are plotted: the mean (90 feeder visits per day) , 1 SD above the mean (150 feeder visits per day), 2 SD above the mean (211 feeder visits per day), and 3 SD above the mean (271 feeder visits per day). These values were chosen because we had a sufficient subset of birds that were observed across all these values of total daily feeder use. Analyses included 50 unique birds (Table S4). However, a subset of 6 birds is provided for illustrative purposes. These individuals were chosen because they were observed across a wide range of total daily feeder visits.	15](#_Toc238649691)

[Figure S3: Illustration of the among-individual differences of the effect of increasing total daily feeder visits on diurnal patterns hourly proportion of total feeder visits. Note that analyses were carried out using continuous variation in total daily feeder visits per bird, however predictions for 3 levels of daily feeder use are plotted: however, for illustrative purposes, model predictions for 4 levels of daily feeder use are plotted: the mean number of feeder visits per day, 1 SD, 2 SD, and 3 SD above the mean. These values were chosen because we had a sufficient subset of birds that were observed across all these values of total daily feeder use. Analyses included 50 unique birds (Table S5). However, a subset of 6 birds is provided for illustrative purposes. These individuals were chosen because they were observed across a wide range of total daily feeder visits.	16](#_Toc238649692)

[Supplementary References	17](#_Toc238649693)

# Text S1: Data filtering and processing

All statistical analyses and RFID data processing were executed using the R Statistical Environment version 4.4.1 [1] and the RStudio interface [2]. The raw RFID data was filtered and processed in several steps. The raw data before filtering and processing was comprised of 210,281 lines of data.

First, we removed data denoting observer visits (N = 144 lines of data). Whenever an observer visited feeders for maintenance, such as retrieving SD cards, replacing batteries and/or refilling seeds, we used an observer PIT tag to log the visit. Observer visits were logged because this allowed us to verify that the internal clocks of the circuit boards were accurate. This left us with 210,137 lines of data.

Second, misread tag IDs (i.e., IDs that did not correspond to any ID in our database and which can arise via signal interference) were removed. This totaled to 5 erroneous tag IDs with 1 visit each representing 0.002% of the total data, or 5 lines of data out of 210,137 reads.

Third, we condensed the RFID data to unique feeder visits. When birds visit the feeders, the same visit can produce multiple lines of data if there is an interruption in the RFID signal, such as if the bird hops on and off the antenna. Based on video recordings of colour banded birds at the feeders in our population, we know that the same individual never returns to the feeder within 12 seconds of leaving [3]. As such, all consecutive RFID detections of the same bird at the same feeder within 12 seconds of each other were collapsed to a single visit, which resulted in the removal of N = 80,960 lines of data. This left us with N = 129,177 lines of data each corresponding to a unique feeder visit.

Fourth, we removed data from 31 July 2025, since feeders were installed midday, preventing us from estimating total daily feeder visits for that day. This did not result in the exclusion of any bird ids from the dataset, as the single bird (N = 1) that discovered the feeder on 31 July 2025 was also detected at the feeders on subsequent days. Removal of July 31^st^, 2025 from the data set left us with N = 129,156 feeder visits.

Fifth, we removed data that was deemed unreliable due to circuit board issues. Specifically, there were intermittent circuit board issues at one feeder location (Feeder 14). Note, only 8 feeder sites were used in this study, although feeder site names include numbers > 8 (see ESM Figure S1). Due to the intermittent circuit board issues at Feeder 14, we removed all data from birds that used Feeder 14 at any point during the experiments. This resulted in the removal of visits N = 4,193 by N = 8 birds. Additionally, Feeder 16 stopped working mid-day on 9 September 2025, presumably after being damaged by a moose (*Alces alces*), but had otherwise no previous issues. Therefore, for the last three days only (9 September 2025 – 11 September 2025), we removed all data from birds that used Feeder 16 at any point in the experiment (N = 456 visits, by N = 10 birds) as we may not have captured all their feeder visits once Feeder 16 failed. However, data from those birds up to and including 8 September 2025 was retained. Finally, we excluded N =1 tag ID that did correspond to a chickadee in our database, but which only visited the feeders once during the study period (31 August 2026). Thus, our final dataset was comprised of N = 124,507 visits by N = 50 birds from August 1^st^, 2025 through September 11^th^, 2025.

# Text S2: Testing for bimodality

For both response variables (visits per hour and proportion of total visits per hour), we first evaluated whether diurnal foraging was best explained by a flat (cathemeral), unimodal, or bimodal distribution. To do this, we constructed 3 separate models for each response variable (i.e., 6 models total) representing each of these distributions, and compared them using Akaike information criterion (AIC). Fixed effects were as follows: an intercept-only model (cathemeral), first-order harmonic sine and cosine terms of time (unimodal), and both first- and second-order harmonic sine and cosine terms of time (bimodal) [4]. For all models, we included individual ID and date as random effects. For both response variables (visits per hour and proportion of total visits per hour), the bimodal model was best supported (see ESM Table S1)

# Text S3: Calculation of daily energy requirements

Daily minimum energy requirements of chickadees can be estimated using Equation 1, which was derived from work by Howitz [5] looking at daily energy intake in chickadees in an outdoor aviary across a range of ambient temperatures.

(Equation 1)	ER = 76.5 kJ – 1.48 kJ/^o^C * T,

Where T is the average daily temperature in degrees Celsius.

In our study, where average daily ambient temperature was ~15^o^C, this corresponds to 54.3 kJ per day. Given that the average metabolizable energy from a sunflower seed is 23.87 kJ per gram (kernel only) (<https://fdc.nal.usda.gov/food-details/2515381/nutrients>), and 1 seed kernel weighs on average 0.05 g (calculated from seeds used for experiments), this corresponds to a minimum requirement of 45 sunflower seeds per day to meet daily energy requirements at ambient temperatures of circa 15 ^o^C. Given the range of temperatures observed over the course of the 6-week study period, we also estimated the expected intake at the lowest observed temperature (minimum overnight temperature = 2.45 ^o^C), and the highest observed temperatures (maximum daytime temperature = 25.17 ^o^C), which corresponds to 61 sunflower seeds and 33 sunflower seeds, respectively, a difference of 28 sunflower seeds across the entire temperature gradient. Note the energetic content per gram of sunflower seed kernel was provided in kcal by the USDA (<https://fdc.nal.usda.gov/food-details/2515381/nutrients>). Values were multiplied by 4.184 to obtain kJ.

# Text S4: Post hoc tests for random slopes

Analysis of diurnal foraging patterns as a function of total daily feeder visits indicated that, at the population level, chickadees increased feeder use throughout the entire day as total daily feeder visits increased, which we assume reflect increases in caching effort. This is in contrast to theoretical models, which predict intense caching to have a single peak [6-9]. At least one earlier empirical study found that in a captive flock of three black-capped chickadees, high levels of caching throughout the day resulted from each individual having a peak in caching at a different time [10]. To evaluate whether this might be occurring in our population as well, we conducted post-hoc analyses where we allowed for individual differences in the diurnal foraging patterns in response to increased total daily feeder visits (i.e., random slopes model). To reduce model complexity and computation time, we did not estimate covariances between random intercepts and random slopes.

Output from random slopes models for hourly foraging and proportion foraging per hour are presented in Tables S4 and S5 respectively. In both cases, models with random slopes (Table S4 and S5) had a significantly better fit than models without random slopes (i.e., models presented in the main text) (Table S6), indicating strong support for individual differences in diurnal foraging patterns in response to increased total daily feeder visits. Plots for a subset of 6 birds are provided in Figures S2 (hourly feeder visits) and S3 (proportion of total daily visits in each hour) for illustrative purposes. These 6 birds were selected based on having the highest daily feeder visits in the dataset, and incidentally, also had daily visits as low as the mean. As such, they had observed daily feeder visits spanning the range of values plotted in Figure S2.

# Table S1: Model comparisons for hourly feeder detections and proportion of total feeding. For each response variable, we evaluated whether the diurnal pattern of foraging was best modelled using a flat (cathemeral), unimodal, or bimodal distribution. For both response variables, the diurnal patters were best explained by the bimodal model (lowest AIC value).

|  |  | Feeder visits per hour | |  | Hourly proportion of  total daily feeder visits | |
| --- | --- | --- | --- | --- | --- | --- |
| Model name | Model fixed effects | AIC | ΔAIC |  | AIC | ΔAIC |
| Cathemeral | ~1 | 129821.6 | 25346.3 |  | 1577.3 | 22939.4 |
| Unimodal | ~cos(2*π*Time/24) + sin(2*π*Time/24) | 112474.0 | 7998.7 |  | -14003.6 | 7358.5 |
| Bimodal | ~cos(2*π*Time/24) + sin(2*π*Time/24) +  cos(2*π*Time/12) + sin(2*π*Time/12) | 104475.3 | 0 |  | - 21362.1 | 0 |

# Table S2: Full model output for binomial trigonometric model for diurnal pattern in total hourly feeder visits in interaction with total daily feeder visits. The model was fitted with a Tweedie error distribution, and the model dispersion parameter (Φ) was 3.2.

|  | **Hourly feeder visits** | | | |
| --- | --- | --- | --- | --- |
| **Fixed effects** | **Estimate** | **SE** | **Z value** | **P value** |
| Intercept | -0.65 | 0.05 | -13.74 | <0.0001 |
| DFV^1^ | 0.57 | 0.02 | 28.92 | <0.0001 |
| cos(2*π*Time/24) | -3.42 | 0.04 | -85.44 | <0.0001 |
| sin(2*π*Time/24) | 0.37 | 0.04 | 25.27 | <0.0001 |
| cos(2*π*Time/12) | -1.46 | 0.02 | -68.52 | <0.0001 |
| sin(2*π*Time/12) | -0.02 | 0.01 | -1.04 | 0.30 |
| cos(2*π*Time/24):DFV^1^ | 0.13 | 0.03 | 4.44 | <0.0001 |
| sin(2*π*Time/24):DFV^1^ | 0.07 | 0.01 | 5.93 | <0.0001 |
| cos(2*π*Time/12):DFV^1^ | 0.10 | 0.02 | 6.59 | <0.0001 |
| sin(2*π*Time/12):DFV^1^ | 0.07 | 0.01 | 6.64 | <0.0001 |
| **Random effects** | **σ** | | | |
| Tag | 0.066 | | | |
| Date | 0.003 | | | |

1. Total daily feeder visits per bird, centered and standardized using the ‘scale’ function in base R prior to analysis.

# Table S3: Full model output for binomial trigonometric model for diurnal pattern in the proportion of total daily visits within each hour in interaction with total daily feeder visits. The model was fitted with a Tweedie error distribution, and the model dispersion parameter (Φ) was 0.115.

|  | **Hourly proportion of total daily feeder visits** | | | |
| --- | --- | --- | --- | --- |
| **Fixed effects** | **Estimate** | **SE** | **Z value** | **P value** |
| Intercept | -4.88 | 0.03 | -192.11 | <0.0001 |
| DFV^1^ | 0.12 | 0.02 | 4.91 | <0.0001 |
| cos(2*π*Time/24) | -3.57 | 0.04 | -88.67 | <0.0001 |
| sin(2*π*Time/24) | 0.35 | 0.01 | 24.16 | <0.0001 |
| cos(2*π*Time/12) | -1.52 | 0.02 | -70.93 | <0.0001 |
| sin(2*π*Time/12) | -0.02 | 0.01 | -1.28 | 0.20 |
| cos(2*π*Time/24):DFV^1^ | 0.19 | 0.04 | 5.15 | <0.0001 |
| sin(2*π*Time/24):DFV^1^ | 0.10 | 0.01 | 6.65 | <0.0001 |
| cos(2*π*Time/12):DFV^1^ | 0.12 | 0.02 | 5.72 | <0.0001 |
| sin(2*π*Time/12):DFV^1^ | 0.09 | 0.01 | 5.95 | <0.0001 |
| **Random effects** | **σ** | | | |
| Tag | 7.82 x10^-11^ | | | |
| Date | 9.61 x10^-11^ | | | |

1. Total daily feeder visits per bird, centered and standardized using the ‘scale’ function in base R prior to analysis.

# Table S4: Full model output for post-hoc test for random slopes in the binomial trigonometric model for diurnal pattern in total hourly feeder visits in interaction with total daily feeder visits. The model was fitted with a Tweedie error distribution, and the model dispersion parameter (Φ) was 2.96.

|  | **Hourly proportion of total daily feeder visits** | | | |
| --- | --- | --- | --- | --- |
| **Fixed effects** | **Estimate** | **SE** | **Z value** | **P value** |
| Intercept | -0.62 | 0.11 | -5.62 | <0.0001 |
| DFV^1^ | 1.01 | 0.08 | 13.212 | <0.0001 |
| cos(2*π*Time/24) | -3.68 | 0.16 | -23.13 | <0.0001 |
| sin(2*π*Time/24) | 0.43 | 0.05 | 9.31 | <0.0001 |
| cos(2*π*Time/12) | -1.42 | 0.08 | -18.55 | <0.0001 |
| sin(2*π*Time/12) | -0.03 | 0.04 | -0.76 | 0.45 |
| cos(2*π*Time/24):DFV^1^ | 0.12 | 0.04 | 3.41 | <0.0001 |
| sin(2*π*Time/24):DFV^1^ | 0.10 | 0.02 | 4.70 | <0.0001 |
| cos(2*π*Time/12):DFV^1^ | 0.13 | 0.02 | 5.72 | <0.0001 |
| sin(2*π*Time/12):DFV^1^ | 0.12 | 0.02 | 6.99 | <0.0001 |
| **Random effects** | **σ** | | | |
| Tag | 0.13 | | | |
| Tag:cos(2*π*Time/24) | 0.25 | | | |
| Tag:sin(2*π*Time/24) | 0.08 | | | |
| Tag:cos(2*π*Time/12) | 0.06 | | | |
| Tag:sin(2*π*Time/12) | 0.06 | | | |
| DFV^1^ | 0.19 | | | |
| Tag:cos(2*π*Time/24):DFV^1^ | 2.80 x10^-8^ | | | |
| Tag:sin(2*π*Time/24):DFV^1^ | 4.56 x10^-3^ | | | |
| Tag:cos(2*π*Time/12):DFV^1^ | 2.88 x10^-3^ | | | |
| Tag:sin(2*π*Time/12):DFV^1^ | 1.76 x10^-3^ | | | |
| Date | 1.90 x10^-3^ | | | |

# Table S5: Full model output for post-hoc test for random slopes in the binomial trigonometric model for diurnal pattern in the proportion of total daily visits within each hour in interaction with total daily feeder visits. The model was fitted with a Tweedie error distribution, and the model dispersion parameter (Φ) was 0.102.

|  | **Hourly proportion of total daily feeder visits** | | | |
| --- | --- | --- | --- | --- |
| **Fixed effects** | **Estimate** | **SE** | **Z value** | **P value** |
| Intercept | -5.20 | 0.10 | -51.95 | <0.0001 |
| DFV^1^ | 0.14 | 0.14 | 4.63 | <0.0001 |
| cos(2*π*Time/24) | -3.89 | 0.06 | -27.28 | <0.0001 |
| sin(2*π*Time/24) | 0.44 | 0.05 | 7.42 | <0.0001 |
| cos(2*π*Time/12) | -0.44 | 0.08 | -19.07 | <0.0001 |
| sin(2*π*Time/12) | -0.03 | 0.05 | -0.65 | 0.52 |
| cos(2*π*Time/24):DFV^1^ | 0.22 | 0.05 | 4.59 | <0.0001 |
| sin(2*π*Time/24):DFV^1^ | 0.14 | 0.04 | 3.91 | <0.0001 |
| cos(2*π*Time/12):DFV^1^ | 0.18 | 0.03 | 5.85 | <0.0001 |
| sin(2*π*Time/12):DFV^1^ | 0.14 | 0.02 | 6.18 | <0.0001 |
| **Random effects** | **σ** | | | |
| Tag | 0.23 | | | |
| Tag:cos(2*π*Time/24) | 0.35 | | | |
| Tag:sin(2*π*Time/24) | 0.13 | | | |
| Tag:cos(2*π*Time/12) | 0.13 | | | |
| Tag:sin(2*π*Time/12) | 0.08 | | | |
| DFV^1^ | 4.2 x10^-9^ | | | |
| Tag:cos(2*π*Time/24):DFV^1^ | 1.12 x10^-8^ | | | |
| Tag:sin(2*π*Time/24):DFV^1^ | 2.74 x10^-2^ | | | |
| Tag:cos(2*π*Time/12):DFV^1^ | 6.82 x10^-3^ | | | |
| Tag:sin(2*π*Time/12):DFV^1^ | 3.87 x10^-3^ | | | |
| Date | 1.38x10^-10^ | | | |

# Table S6: Model comparison between models without vs. models with random slopes for changes in diurnal foraging patterns as a function of total daily feeder visits. Chi^2^ and p-values provide strength of support for the more complex model structure (i.e., random slopes)

| Response variable | Model | DF | Log Likelihood | Chi^2^ | P-value |
| --- | --- | --- | --- | --- | --- |
| Hourly feeder visits | Random intercepts only | 14 | 102563 |  |  |
|  | Random intercepts and Random slopes | 23 | 100417 | 2237 | <0.0001 |
| Proportion of total feeder visits per hour | Random intercepts only | 14 | 10736 |  |  |
|  | Random intercepts and Random slopes | 23 | 11695 | 1918 | <0.0001 |

# Figure S1. Map of the field site at the University of Alberta Botanic Garden, Alberta, Canada. (53° 24' N, 113° 45' W, 0.97 km^2^). The black dot in the inset map represents the location of the field site within Canada. Garden limits are shown by solid grey lines; dotted grey lines represent walking pathways within the garden. Feeder locations are represented by black circles. West of the garden, the black lines represent Alberta Highway 60. The grey shaded area represents the public visitors’ area, which includes the managed horticulturist gardens but also mixed forest areas. Map created by Josué Arteaga-Torres, and is used with permission.

# Figure S2 : Illustration of the among-individual differences of the effect of increasing total daily feeder visits on diurnal patterns hourly feeder visits. Note that analyses were carried out using continuous variation in total daily feeder visits per bird, however, for illustrative purposes, model predictions for 4 levels of daily feeder use are plotted: the mean (90 feeder visits per day) , 1 SD above the mean (150 feeder visits per day), 2 SD above the mean (211 feeder visits per day), and 3 SD above the mean (271 feeder visits per day). These values were chosen because we had a sufficient subset of birds that were observed across all these values of total daily feeder use. Analyses included 50 unique birds (Table S4). However, a subset of 6 birds is provided for illustrative purposes. These individuals were chosen because they were observed across a wide range of total daily feeder visits.

# Figure S3: Illustration of the among-individual differences of the effect of increasing total daily feeder visits on diurnal patterns hourly proportion of total feeder visits. Note that analyses were carried out using continuous variation in total daily feeder visits per bird, however predictions for 3 levels of daily feeder use are plotted: however, for illustrative purposes, model predictions for 4 levels of daily feeder use are plotted: the mean number of feeder visits per day, 1 SD, 2 SD, and 3 SD above the mean. These values were chosen because we had a sufficient subset of birds that were observed across all these values of total daily feeder use. Analyses included 50 unique birds (Table S5). However, a subset of 6 birds is provided for illustrative purposes. These individuals were chosen because they were observed across a wide range of total daily feeder visits.

# Supplementary References

[1] R Development Core Team. 2024 R: A language and environment for statistical computing. 4.4.1 ed. Vienna, Austria, See <http://www.R-project.org>

[2] R Studio Team. 2020 RStudio: Integrated Development for R. Boston, MA, RStudio, PBC.<http://www.rstudio.com/>

[3] Arteaga-Torres, J.D., Wijmenga, J.J. & Mathot, K.J. 2020 Visual cues of predation risk outweigh acoustic cues: a field experiment in black-capped chickadees. *Proceedings of the Royal Society B: Biological Sciences* **287**, 20202002. (doi:10.1098/rspb.2020.2002).

[4] Iannarilli, F., Gerber, B.D., Erb, J. & Fieberg, J.R. 2025 A ‘how-to’ guide for estimating animal diel activity using hierarchical models. *J. Anim. Ecol.* **94**, 182-194. (<https://doi.org/10.1111/1365-2656.14213>).

[5] Howitz, J.L. 1981 A population study of the black-capped chickadee [Ph.D.]. Minnesota, USA, University of Minnesota.

[6] Brodin, A. 2007 Theoretical models of adaptive energy management in small wintering birds. *Philos. Trans. R. Soc. B* **362**, 1857-1871. (10.1098/rstb.2006.1812).

[7] McNamara, J.M., Houston, A.I. & Krebs, J.R. 1990 Why hoard? The economics of food storing in tits, Parus spp. *Behav. Ecol.* **1**, 12-23. (10.1093/beheco/1.1.12).

[8] Pravosudov, V.V. & Lucas, J.R. 2001 A dynamic model of short-term energy management in small food-caching and non-caching birds. *Behav. Ecol.* **12**, 207-218. (10.1093/beheco/12.2.207).

[9] Pravosudov, V.V. & Lucas, J.R. 2001 Daily patterns of energy storage in food-caching birds under variable daily predation risk: a dynamic state variable model. *Behav. Ecol. Sociobiol.* **50**, 239-250. (10.1007/s002650100361).

[10] Boisvert, M.J. 1999 Winter food-caching, feeding, and body weight in relation to social dominance in black-capped chickadees (*Poecile atricapillus*) [MSc]. London, Ontario, University of Western Ontario.
